# Identifying clusters of people with Multiple Long-Term Conditions using Large Language Models: a population-based study

**DOI:** 10.1038/s41746-025-01806-9

**Published:** 2025-07-17

**Authors:** Alexander Smith, Thomas Beaney, Carinna Hockham, Bowen Su, Paul Elliott, Laura Downey, Spiros Denaxas, Payam Barnaghi, Abbas Dehghan, Ioanna Tzoulaki

**Affiliations:** 1 Department of Epidemiology and Biostatistics, Imperial College London, London, UK; 2 The George Institute for Global Health, Imperial College London, London, UK; 3 Department of Surgery & Cancer, Imperial College London, London, UK; 4 Institute of Health Informatics, University College London, London, UK; 5 Department of Brian Sciences, Imperial College London, London, UK; 6 Biomedical Research Foundation Academy of Athens, Athens, Greece

**Keywords:** Epidemiology, Disease prevention, Diseases, Epidemiology

## Abstract

Identifying clusters of people with similar patterns of Multiple Long-Term Conditions (MLTC) could help healthcare services to tailor care. In this population-based study, we developed a pipeline incorporating a DeBERTa language model to generate gender-specific clusters. Our model, EHR-DeBERTa, was pre-trained on longitudinal sequences of diagnoses, medications and test results from primary care electronic health records of 5.8 million patients in the UK. EHR-DeBERTa was used to generate patient embeddings for males and females separately, and clusters were identified by K-Means. Fifteen clusters were identified in females and seventeen in males, categorized into low disease burden, mental health, cardiometabolic, respiratory and mixed diseases. Cardiometabolic and mental health conditions showed the strongest separation across clusters, with older patients in cardiometabolic clusters. Our approach demonstrates how LLMs can provide interpretable insights into disease patterns. Future work incorporating clinical outcomes could enhance risk prediction and support precision-medicine for people with MLTC.

## Introduction

Worldwide, a growing number of people are living with Multiple Long-Term Conditions (MLTC), a health state characterised by the co-existence of two or more chronic conditions^1,2^. This presents a significant challenge for health systems^3^, as those with MLTC experience worse health outcomes^4^, poorer quality of life^5^ and greater use of health services^6^. Furthermore, health services are often designed around the care of single diseases, resulting in an accumulating burden of clinical appointments for patients and challenges in navigating healthcare^7^.

One of the biggest difficulties in designing interventions to address the challenges of MLTC is clinical heterogeneity. Many different chronic conditions may be included in the definition of MLTC^8^ and there are many possible unique combinations of diseases that may occur in people^9^. As a result, there is growing interest in identifying patterns of MLTC which commonly occur in a population^10^. Clustering offers a data-driven approach to identifying these patterns, which generates groups of similar diseases, or of people based on similar patterns of diseases. Most clustering approaches have been applied to diseases, identifying strong patterns of cardiometabolic and of mental health conditions^11,12^, but increasingly, approaches have been applied to identify clusters of people based on a holistic view of their medical history^13,14^. Understanding groups of people with similar conditions may more directly inform understanding of shared causes and outcomes, and inform health service design, such as designing a service tailored to a specific cluster^13,15^.

Moreover, studies in MLTC have often employed cross-sectional designs, capturing the diseases a person has at a single point in time. However, this does not account for the temporal order of disease development, which has been found to have significant value in predicting healthcare utilisation and clinical outcomes^16^. Use of deep learning models, such as transformers, enable sequences to be incorporated and have been increasingly applied to predictive tasks in healthcare^16–18^. These approaches can be applied to the rich information recorded within electronic health records (EHRs), which include symptoms, clinical examination findings, prescribed medications and laboratory test results, allowing the separation of phenotypes according to these factors and not only on diseases. However, previous models that incorporate diagnostic codes as words, such as BEHRT^17^ and MedBERT^18^, have utilised a limited diagnosis vocabulary, are built on earlier-generation models such as BERT^19^ and, to our knowledge, have not been utilised for patient clustering to explain common patterns of diseases.

In this study, we aimed to generate gender-specific clusters of patients with similar sociodemographic profiles and sequences of clinical information recorded in EHRs, using a large and nationally representative sample of people registered to general practices (GPs) in the United Kingdom (UK). To achieve this, we first develop a transformer architecture which incorporates information on the chronological order of disease development utilising a comprehensive disease and medication vocabulary. We utilise the model to generate vector representations of patient records, called patient embeddings, followed by clustering of the embeddings by K-Means. Finally, we describe the resulting clusters by gender, socio-demographic profiles and diseases to identify common MLTC patterns within the population.

## Results

### Cohort description

A total of 5,846,480 patients registered to GP practices in the Clinical Practice Research Datalink (CPRD) were included in the analysis. The mean (standard deviation (SD)) age was 66 (16) years, 3,201,230 (54.8%) were female and 2,645,250 (45.2%) were male (Table 1). Information on ethnicity was recorded for 37.7% of participants, of whom 2,055,170 (93.4%) were White, 43,345 (2.0%) Black, 63,972 (2.9%) South Asian, 11,447 (0.5%) Mixed and 26,097 (1.2%) of ‘Other’ ethnicity. Relatively more patients were living in less deprived areas, with 22.6% in the least deprived and 15.8% in the most deprived areas. The median number of events recorded in the longitudinal sequences was 24 across the entire cohort.

**Table 1:** Characteristics of the study population

	Female	Male	Total
*N*	3,201,230 (54.8%)	2,645,250 (45.2%)	5,846,480
Age (Median, IQR)	67 (54–81)	66 (54–77)	66 (54–79)
Ethnicity (*n*, %)	1,189,387 (37.2%)	1,010,644 (38.0%)	2,200,031 (37.7%)
White	1,110,639 (93.4%)	944,531 (93.5%)	2,055,170 (93.4%)
Black	24,536 (2.1%)	18,809 (1.9%)	43,345 (2.0%)
South Asian	33,212 (2.8%)	30,760 (3.0%)	63,972 (2.9%)
Mixed	6,446 (0.5%)	5001 (0.5%)	11,447 (0.5%)
Other	14,554 (1.2%)	11,543 (1.1%)	26,097 (1.2%)
Socioeconomic deprivation by Index of Multiple Deprivation (*n*, %)	1,638,451 (51.2%)	1,359,107 (51.4%)	2,997,558 (51.3%)
1 (Least deprived)	372,275 (22.7%)	304,134 (22.4%)	676,409 (22.6%)
2	366,841 (22.4%)	299,736 (22.1%)	666,577 (22.2%)
3	347,845 (21.2%)	285,278 (21.0%)	633,123 (21.1%)
4	299,559 (18.3%)	248,905 (18.3%)	548,464 (18.3%)
5 (Most deprived)	251,931 (15.4%)	221,054 (16.3%)	472,985 (15.8%)

### Disease patterns within clusters

**Fig. 1:**
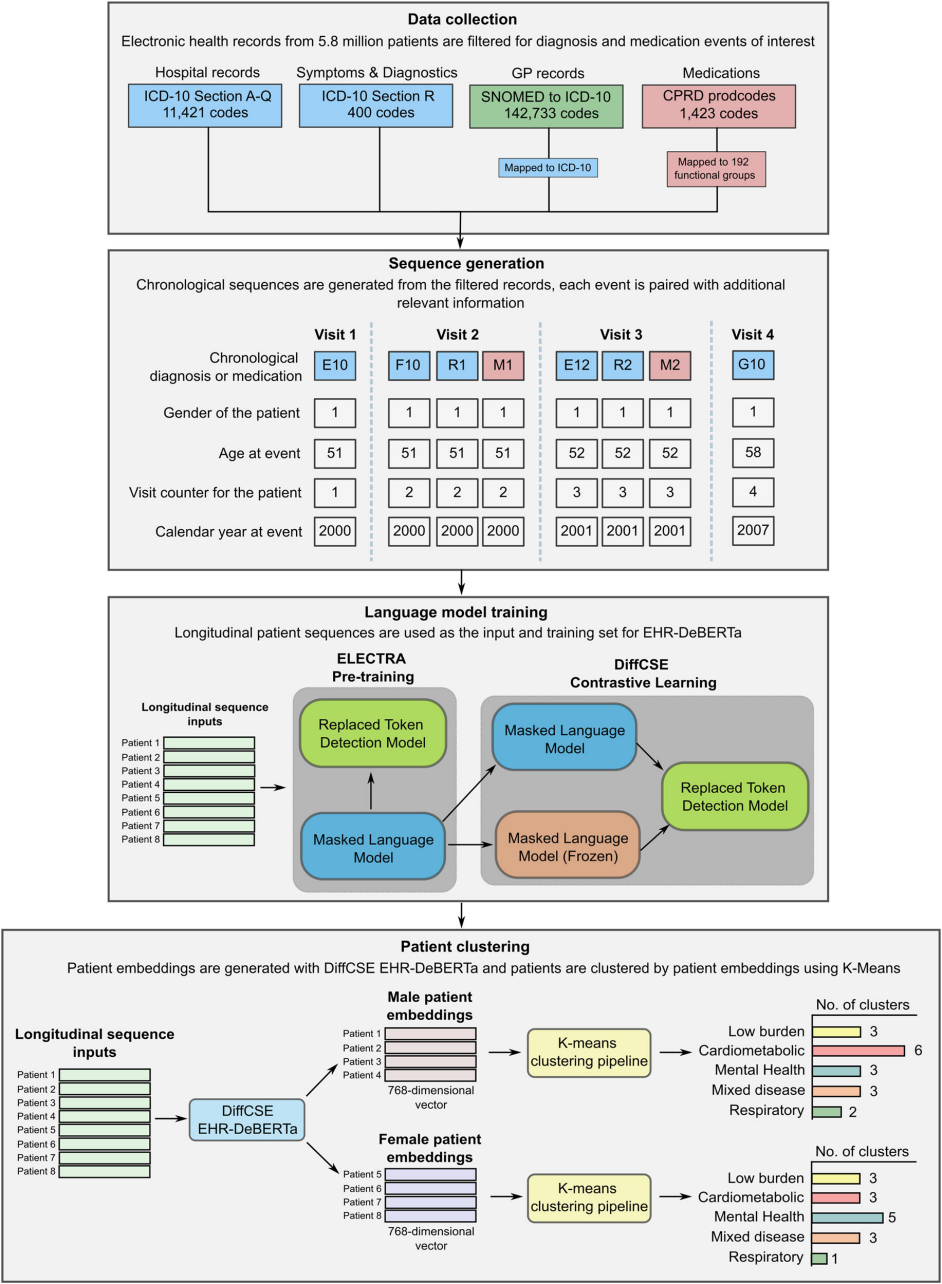
Study overview. The study overview of training the large language model and generating patient clusters using EHRs.

We developed a pipeline incorporating a Large Language Model (LLM) which learns sequences of clinical diagnoses (including 54 chronic conditions and three risk factors), symptoms, medications and laboratory test results to output a quantitative vector representation. Based on 197 clinically established disease co-morbidity pairs, there was a high degree of similarity between the disease embeddings of each pair (median cosine similarity = 0.58 across all pairs), demonstrating the model’s ability to learn clinically meaningful relationships between conditions (Supplementary Table 4). This vector representation was used as an input to generate gender-specific clusters (see Fig. 1 and ‘Methods’). We identified an optimal number of 15 clusters in females and 17 clusters in males. These clusters were stable across subsamples of our cohort, with an average cosine distance between K-Means centroids of 0.15 and 0.10 when using 25% of the population across 100 replicates in females and males, respectively (Supplementary Table 6). The clusters were also stable across time for both genders, with an average cluster cohesion difference of 0.02 and 0.01 between samples assigned to each cluster using their entire medical history (final clusters) and age-spliced medical histories (over time) for females and males respectively.

**Fig. 2:**
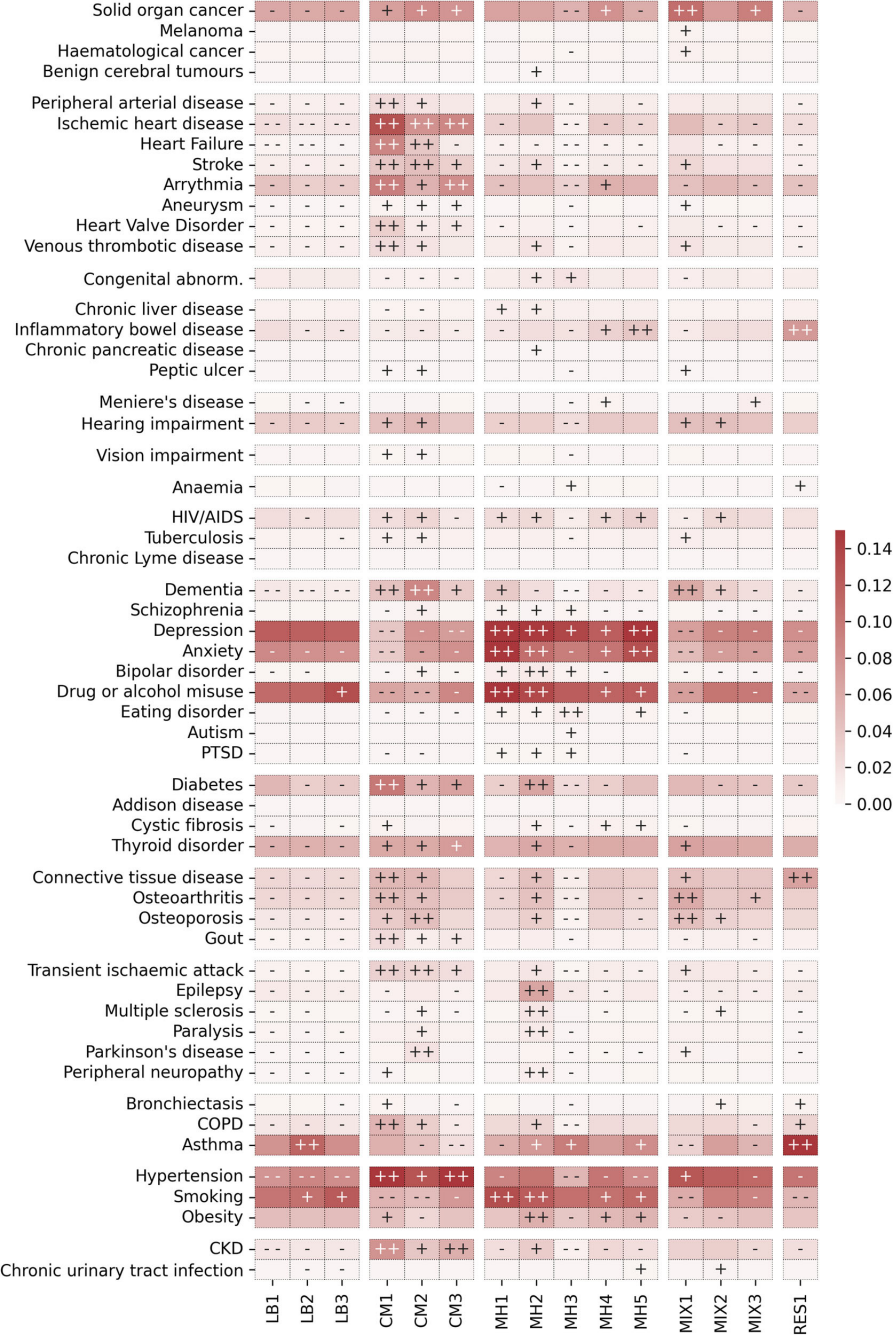
Disease prevalence within female clusters. The disease prevalence across clusters of 3,201,230 females from CPRD. The heatmap colour indicates the cluster-weighted frequency (c-DF-IPF) of each LTC, with red indicating higher frequency. The symbols within each cell are indicators of the Z-score difference between observed and expected disease frequency from 1-vs-all Chi-squared testing (++ ≥ 50, 50> + ≥10, - - ≤ −50, −50< − ≤−10). LB low burden, CM cardiometabolic, MH mental health, MIX mixed, RES respiratory.

**Fig. 3:**
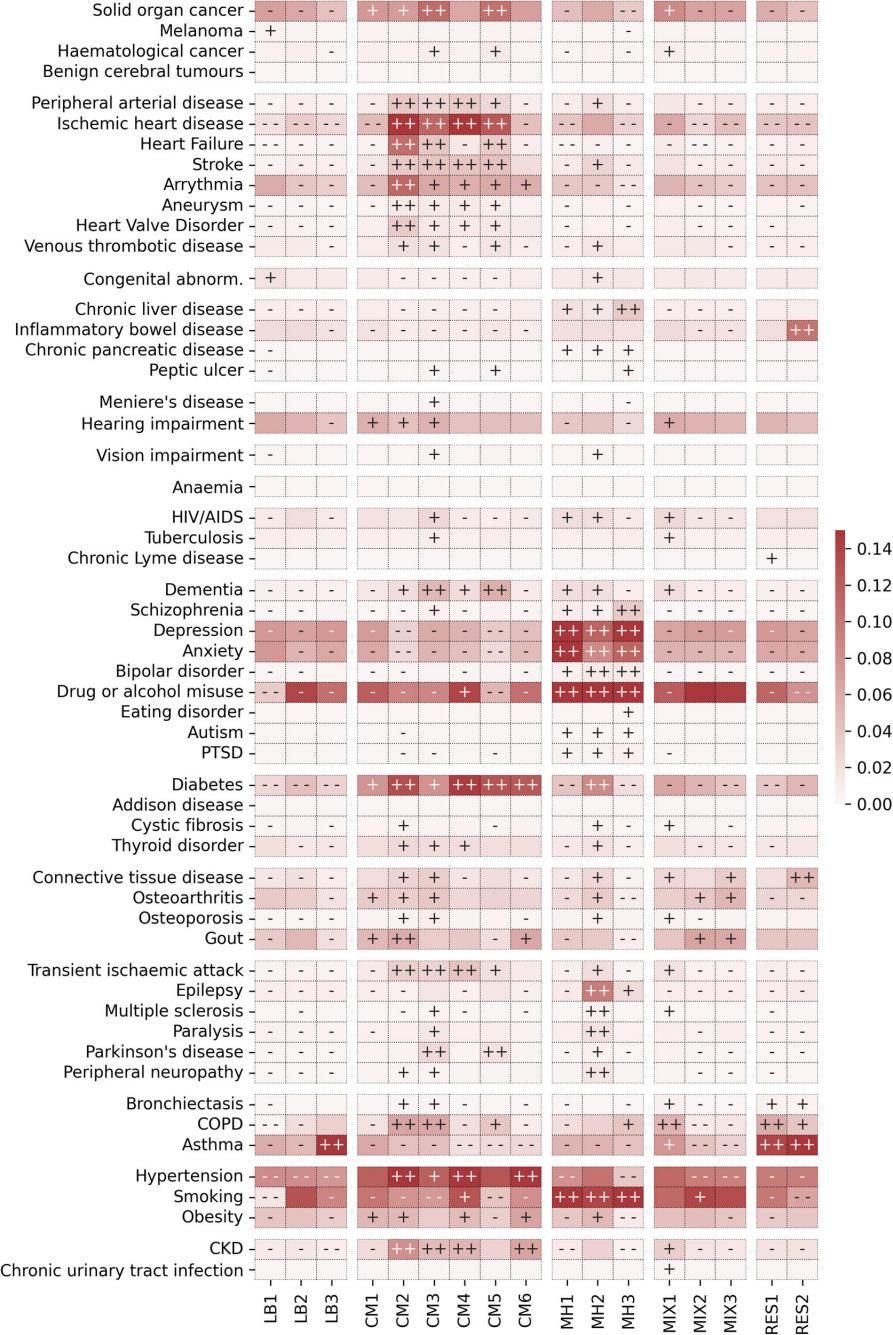
Disease prevalence within male clusters. The disease prevalence across clusters in 2,645,250 males from CPRD. The heatmap colour indicates the cluster-weighted frequency (c-DF-IPF) of each LTC, with red indicating higher frequency. The symbols within each cell are indicators of the Z-score difference between observed and expected disease frequency from 1-vs-all Chi-squared testing (++ ≥ 50, 50> + ≥10, - - ≤ −50, −50< − ≤−10). LB low burden, CM cardiometabolic, MH mental health, MIX mixed, RES respiratory.

Cardiovascular and metabolic diseases, mental health conditions and risk factors including hypertension, smoking and obesity varied significantly in prevalence across clusters in both females (Fig. 2) and males (Fig. 3). Few diseases showed little variation across clusters. For example, there was no statistically significant difference in the prevalence of Addison disease across clusters in males or females. Following assessment, we assigned these manually to five broad groupings of the 15 female and 17 male clusters characterised by the following patterns: (i) low disease burden (characterised lower than expected prevalence in all but 2 diseases); (ii) mental health; (iii) cardiometabolic diseases; (iv) respiratory diseases and (v) mixed diseases.

In females (Fig. 2), three clusters of relatively healthy individuals with low disease burden (LB) were found, representing 20.3% of the female population. One had low prevalence across all diseases and risk factors (LB1) and two others were characterised by high prevalence of smoking and either alcohol or drug misuse or asthma alone (LB2 and LB3). Of three cardiometabolic (CM) clusters (18.8% of females), one had very high prevalence across all cardiovascular, kidney and metabolic conditions along with respiratory disease and gout (CM1), another was characterised by a high prevalence of cardiometabolic and neurological conditions including dementia and Parkinson Disease (CM2), and a third had high prevalence of ischaemic heart disease and renal disease but lower prevalence of heart failure (CM3). All cardiometabolic clusters had lower prevalence of mental health conditions. Overall, 35.6% of females were in five subtypes of mental health clusters, one with marked high prevalence (MH1) of mental health conditions, smoking and drug or alcohol misuse, one with high prevalence of multiple sclerosis and other neurological conditions (MH2), one with higher prevalence of eating disorders and lower prevalence of cardiovascular disease and risk factors (MH3), one with relatively intermediate prevalence of anxiety depression and a range of diverse conditions (MH4), and finally another by higher prevalence of inflammatory bowel disease (IBD) (MH5). Finally, three mixed condition clusters (21.3% of females) were characterised by cancer, dementia, osteoarthritis and osteoporosis (MIX1), dementia, hearing impairment, osteoporosis and multiple sclerosis (MIX2), and cancer, osteoarthritis and Meniere’s Disease (MIX3).

In males (Fig. 3), 14.6% were assigned to one of three low disease burden clusters, one being low in all conditions except congenital disease and melanoma (LB1), another characterised by low prevalence of all conditions (LB2) and another with a high prevalence of asthma alone (LB3). Overall, 34.6% of males were assigned to one of six cardiometabolic clusters. One had higher prevalence of diabetes, gout and obesity (CM1), and another had higher prevalence of diabetes, hypertension and Chronic Kidney Disease (CKD) (CM6). The remaining four cardiometabolic clusters (CM2-CM5) had high prevalence of cardiovascular diseases. Of these, CM2 had particularly high prevalence of cardiovascular conditions, as well as Chronic Obstructive Pulmonary Disease (COPD). CM4 was also characterised by a high prevalence of cardiovascular diseases, but in contrast to CM2, a low prevalence of heart failure and COPD. CM3 and CM5 had an intermediate prevalence of cardiovascular conditions, cancer, dementia and Parkinson’s disease, but CM3 had high prevalence of CKD, COPD and other neurological diseases which were not higher than expected in CM5. As in females, the cardiometabolic clusters had lower prevalence of mental health conditions. Three mental health clusters (MH1, MH2 and MH3, representing 19.9% of males) each had a high prevalence of anxiety, depression, drug or alcohol misuse and smoking, but MH2 also had high prevalence of neurological conditions and MH3 had high prevalence of schizophrenia, bipolar disorder and chronic liver disease. Three mixed condition clusters (21.5% of males) were found: one including cancer, respiratory disease and infectious diseases (MIX1), another including gout, osteoarthritis and smoking (MIX2) and another including connective tissue disease, gout and osteoarthritis (MIX3). Finally, 9.5% of males were assigned two respiratory clusters: one characterised by respiratory diseases including asthma, bronchiectasis and COPD (RES1) and another which also included connective tissue disease and IBD (RES2).

### Demographic differences between clusters

Between genders, clusters within the broad groupings correlated well, for example, cardiometabolic or respiratory clusters (Supplementary Fig. 2). In some cases, a single cluster in one gender was similar to multiple clusters in the other. For example, the female low burden clusters were similar to male low burden clusters but also shared similarities with male mental health and mixed disease clusters.

The age distributions within each cluster were relatively wide, but with notable, differences in the age distributions between clusters. People assigned to cardiometabolic clusters were older (according to their age at their last record) than those in the low disease burden or mental health clusters (Table 2 and Supplementary Fig. 1). The mixed and respiratory clusters had more uniform age distributions reflecting a wider age range of individuals within these clusters compared to other clusters (Table 2 and Supplementary Fig. 1).

**Table 2:** Description of patient clusters, size (N) and median age (age of individuals at the time of their last record) and follow-up time in female and male CPRD participants

Cluster	Cluster description	*N* (%)	Median age (IQR), years	Median follow-up (IQR), years
Females				
Low disease burden (LB)		650,897 (20.3)	57 (49–70)	27 (19–38)
LB1	Low disease burden and low prevalence of risk factors	291,665 (9.1)	57 (48–70)	28 (19–39)
LB2	Low disease burden, high prevalence of smoking and asthma	190,005 (5.9)	59 (50–70)	27 (18–39)
LB3	Low disease burden, high prevalence of smoking/alcohol or drug misuse	169,227 (5.3)	57 (49–68)	27 (18–37)
Cardiometabolic (CM)		601,704 (18.8)	83 (74–89)	30 (21–42)
CM1	Multiple cardiovascular conditions, COPD, CKD, hypertension, diabetes, gout, dementia	240,443 (7.5)	85 (78–90)	29 (18–45)
CM2	Multiple cardiovascular conditions, dementia, Parkinson’s disease	194,004 (6.1)	85 (76–91)	28 (16–43)
CM3	Ischaemic heart disease, hypertension, CKD, arrythmia	167,257 (5.2)	76 (66–85)	29 (18–44)
Mental health (MH)		1,138,738 (35.6)	60 (51–74)	30 (21–42)
MH1	Multiple mental health conditions, high prevalence of smoking/alcohol or drug misuse	246,272 (7.7)	64 (54–76)	30 (21–42)
MH2	Multiple mental health conditions, epilepsy, multiple sclerosis and other neurological conditions, smoking & obesity	240,490 (7.5)	67 (56–79)	34 (24–46)
MH3	Eating disorders, several mental health conditions, low prevalence of cardiovascular conditions and risk factors	239,858 (7.5)	50 (45–56)	26 (18–36)
MH4	Anxiety, depression and mixed conditions of intermediate prevalence	226,129 (7.1)	66 (55–78)	31 (22–44)
MH5	Anxiety, depression and IBD	185,989 (5.8)	60 (51–74)	31 (23–42)
Mixed (MIX)		683,135 (21.3)	72 (60–83)	29 (19–43)
MIX1	Cancer, dementia, osteoarthritis, osteoporosis	250,093 (7.8)	76 (67–85)	28 (17–43)
MIX2	Dementia, hearing impairment, osteoporosis, multiple sclerosis	241,945 (7.6)	69 (57–81)	31 (21–44)
MIX3	Cancer, osteoarthritis, Meniere’s disease	191,097 (6.0)	68 (56–80)	28 (18–41)
Respiratory (RES)		126,756 (4.0)	64 (53–76)	29 (20–42)
RES1	Asthma, COPD, IBD and connective tissue disease	126,756 (4.0)	64 (53–76)	29 (20–42)
Males				
Low disease burden		386,484 (14.6)	57 (49–68)	28 (18–41)
LB1	Low disease burden and low prevalence of risk factors, except congenital disease and melanoma	140,398 (5.3)	57 (50–67)	30 (20–43)
LB2	Low disease burden	129,687 (4.9)	59 (50–70)	27 (18–39)
LB3	Low disease burden, high prevalence of asthma	116,399 (4.4)	56 (48–67)	27 (17–40)
Cardiometabolic		914,067 (34.6)	74 (64–83)	27 (17–41)
CM1	Diabetes, gout, obesity	209,963 (7.9)	65 (55–76)	29 (20 –42)
CM2	Multiple cardiovascular conditions, COPD, CKD, diabetes, gout	170,306 (6.4)	80 (72–86)	28 (18–42)
CM3	Multiple cardiovascular conditions, cancer, COPD, CKD, dementia, Parkinson’s disease	155,216 (5.9)	81 (72–87)	28 (17–42)
CM4	Multiple cardiovascular conditions (except heart failure), CKD, diabetes	154,298 (5.8)	71 (63–79)	27 (18–41)
CM5	Multiple cardiovascular conditions, cancer, diabetes, dementia, Parkinson’s disease	113,398 (4.3)	77 (68–85)	21 (12–33)
CM6	Diabetes, hypertension, CKD	110,886 (4.2)	69 (60 –77)	26 (17–40)
Mental health		525,461 (19.9)	59 (51–70)	29 (20 –41)
MH1	Multiple mental health conditions, high prevalence of smoking/alcohol or drug misuse	204,626 (7.7)	58 (50–68)	28 (19–40)
MH2	Multiple mental health conditions, drug or alcohol misuse, epilepsy and other neurological conditions, smoking	189,899 (7.2)	66 (56–76)	31 (22–44)
MH3	Multiple mental health conditions, including schizophrenia, high prevalence of smoking/alcohol or drug misuse and chronic liver disease	130,936 (4.9)	54 (47–62)	27 (18–39)
Mixed		567,472 (21.4)	65 (54–76)	28 (19–41)
MIX1	Cancer, infectious disease and respiratory disease	241,920 (9.1)	71 (59–81)	30 (20–44)
MIX2	Gout, osteoarthritis, smoking	166,641 (6.3)	60 (51–72)	28 (19–40)
MIX3	Connective tissue disease, gout, osteoarthritis	158,911 (6.0)	62 (52–72)	26 (17–38)
Respiratory		251,766 (9.5)	63 (52–74)	29 (20–42)
RES1	Asthma, bronchiectasis and COPD	132,836 (5.0)	63 (53–74)	29 (19–42)
RES2	Asthma, bronchiectasis, COPD, IBD and connective tissue disease	118,930 (4.5)	62 (52–74)	29 (20–42)

*CKD* Chronic Kidney Disease, *COPD* Chronic Obstructive Pulmonary Disease, *IBD* Inflammatory Bowel Disease.

**Fig. 4:**
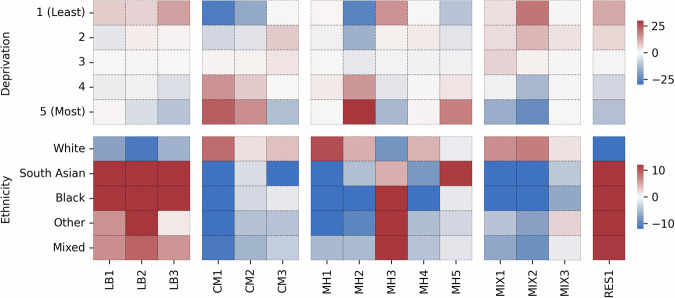
Ethnicity and deprivation associations across female clusters. Heatmap colours indicate the Z-scores differences in observed and expected frequency of each ethnicity and deprivation quantile with red indicating higher frequency (overrepresentation) and blue indicating lower frequency (underrepresentation), based on 1-vs-all Chi-squared testing. LB low burden, CM cardiometabolic, MH mental health, MIX mixed, RES respiratory.

**Fig. 5:**
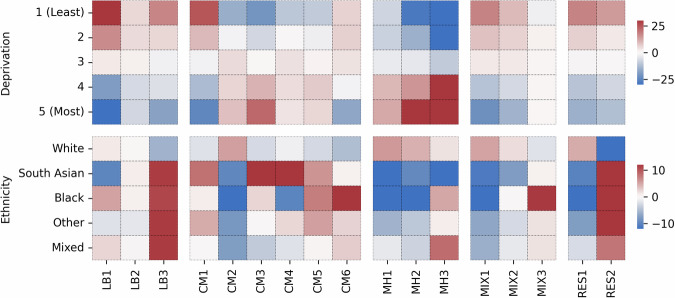
Ethnicity and deprivation associations across male clusters. Heatmap colours indicate the Z-scores differences in observed and expected frequency of each ethnicity and deprivation quantile with red indicating higher frequency (overrepresentation) and blue indicating lower frequency (underrepresentation), based on 1-vs-all Chi-squared testing. LB low burden, CM cardiometabolic, MH mental health, MIX mixed, RES respiratory.

There were also significant differences in ethnicity and deprivation distributions across clusters (Figs. 4, 5 and Supplementary Table 5). For example, within the male cardiometabolic clusters, distinct subgroups emerged: CM2 had an overrepresentation of white individuals not living in deprived areas, CM1 was predominantly composed of South Asian individuals not living in deprived areas and CM6 had a higher proportion of Black individuals living in deprived areas. Similarly, notable disparities were observed in respiratory clusters (RES1 in females and RES2 in males), which were overrepresented by non-white individuals living in areas with low deprivation. We also found that the low disease burden clusters in both males and females were underrepresented by people living in the most deprived areas.

### Latent class analysis patient clusters

As a comparison, we alternatively used Latent Class Analysis (LCA) to cluster patients. The optimum number of classes based on Akaike information criterion (AIC) and Bayesian information criterion (BIC) on both the male and female cohorts was 8 and full class loadings can be found in Supplementary Table 11. When using the same number of clusters as the LLM pipeline, LCA generated highly variable cluster sizes (Supplementary Table 10), with most of the population characterised by the three largest clusters in both males (58.99%) and females (61.53%). The largest male cluster comprised 31.41% of the cohort and was defined by asthma, depression and hypertension. In the female population, the largest cluster contained 30.27% of the population, defined by asthma, depression and anxiety.

Based on the top three LTCs for each cluster, many of the male clusters shared the same conditions, with 8 out of 17 clusters containing both smoking and drug or alcohol misuse, 10 clusters containing hypertension and 5 containing depression. One of the male clusters also had no meaningful loadings and contained only a single individual. Similar overlap was found within female clusters, with 10 out of 15 clusters containing hypertension, 8 containing depression and many other conditions such as anxiety and diabetes common amongst at least three clusters.

## Discussion

Using a nationally representative dataset of nearly 6 million UK individuals, we applied LLM-based clustering to group patient trajectories based on disease progression over time. A key innovation of our method is the use of patient embeddings generated by an LLM to cluster patients rather than diseases, leveraging the full spectrum of clinical information – including prescriptions, diagnoses and laboratory tests – representing a major advance on earlier work that has primarily mapped disease co-occurrence cross-sectionally. By employing an advanced transformer architecture (EHR-DeBERTa), our approach facilitates a dynamic and more personalised understanding of MLTC patterns, resulting in interpretable gender-specific patient clusters. Five main patient clusters emerged: healthy young individuals, middle-aged individuals with predominant mental health conditions, older patients with cardiometabolic diseases, respiratory disease clusters and mixed multimorbidity clusters across body systems. Within each broad group, there were different cluster subtypes representing distinct patient trajectories including recognisable co-occurrence of linked conditions as well as other less well-studied disease combinations. Findings confirm that mental health conditions peak in midlife, while cardiometabolic diseases are more prevalent in older adults, reinforcing the need for age-specific healthcare strategies. This age-related variation highlights the need for tailored healthcare strategies that address the evolving burden of MLTCs over the life course and emphasises the importance of early interventions before disease has occurred, for example, to prevent transitions from a low disease burden cluster to a mental health cluster in midlife or a cardiometabolic cluster later in life.

Mental health clusters were more common in females (28% vs. 13% in males), aligning with known higher depression and anxiety prevalence in women^20^. In both genders, we identified a highly comorbid cluster with multiple mental health conditions, co-occurring with diabetes, obesity and neurological conditions including Parkinson’s Disease and multiple sclerosis^21,22^. These findings reinforce existing evidence on bidirectional associations between these conditions^23^ and further stresses the need for integrated management approaches that address both mental and physical health within a unified care framework.

We observed significant variation in the prevalence of cardiometabolic conditions across clusters, suggesting that these conditions play a dominant role in distinguishing patient phenotypes. Cardiometabolic clusters were more prevalent in males compared to females and females in cardiometabolic clusters tended to be older than their male counterparts, consistent with literature^24,25^. However, the composition of clusters showed notable similarities between genders. In both males and females, we observed a highly comorbid cluster of cardiovascular, metabolic and renal comorbidities, often co-occurring with other linked conditions such as gout, COPD and neurodegeneration. This underscores the well-recognised bidirectional association between the dysfunction of the heart, kidneys and metabolic system and their systemic multi-organ consequences^26^. The co-occurrence of neurodegeneration highlights an important heart-brain-liver axis which is less well-characterised. As more therapeutic options emerge, it is crucial to develop integrated treatment strategies that maximise multi-organ benefits and establish shared guidelines for managing these interconnected conditions.

Interestingly, mental health and cardiovascular clusters were mutually exclusive: clusters with a high prevalence of cardiometabolic conditions had a lower prevalence of mental health conditions and vice versa. This contrasts with strong epidemiological evidence of increased incidence of cardiovascular disease in those with pre-existing mental health conditions, particularly psychotic disorders^27^. Possible explanations include the younger age of those in the mental health clusters, who may not yet have developed cardiovascular disease and the older age of those in the cardiometabolic clusters, mental health conditions may be underdiagnosed due to stigma and barriers to accessing support^28^.

Other multi-organ effects were evident from the patient clustering. For example, the clustering of patients with IBD with COPD and other respiratory conditions. These conditions may share similar underlying pathways such as infection, immune activation, inflammation and shared microbiota effects which may lead to common treatments and monitoring of patients with IBD for the potential of developing respiratory disease^29^. Similarly, the association between osteoporosis and dementia raises the possibility of an underlying biologic mechanism linking bone loss and cognitive decline, especially in females^30^.

Within each broader cluster, individuals with similar ethnic backgrounds and socioeconomic status tended to cluster together. For example, we identified distinct male cardiometabolic clusters with an overrepresentation of White individuals (CM2), South Asian individuals (CM1) and Black individuals (CM6), with the latter two clusters having a lower median age than CM2. We also found that low disease burden clusters were underrepresented by people living in more deprived areas. Several conclusions can be drawn. Firstly, MLTCs are a widespread issue across the population and although not confined to populations with specific ethnic or socioeconomic backgrounds, people in low disease burden clusters are more likely to be from less deprived areas. Secondly, individuals with similar socioeconomic status and ethnic backgrounds may experience comparable trajectories of comorbidities, reflecting shared risk factors within these groups. Thirdly, clusters including people living in areas of greater deprivation and from non-White ethnic backgrounds tend to be younger, possibly indicating earlier onset of MLTCs in these subgroups. Previous research has also highlighted that people living in more deprived areas have a faster accumulation of conditions and higher mortality rates^31^. Collectively, our findings emphasise that tailored healthcare strategies are essential to address the diverse needs of different population groups in addition to integrated approaches to managing MLTC.

Our study found a larger number of clusters than in many studies in MLTC research^11^. While a higher number of clusters will create more homogeneous clusters, this should be balanced against the increasing cognitive complexity needed to understand the clusters, which may impact on their application for direct clinical tasks. For this reason, we grouped clusters into broader categories, which allows a multi-resolution view of clusters, where users can move between finer or coarser representations depending on the task^32^. The ability for our LLM-based pipeline to identify a large number of clinically meaningful clusters is a major advantage over other analytical methods, such as LCA, that have limited ability to jointly model longitudinal information and paired covariates, overlooking the complexity and variability of patient profiles. This is highlighted by the largely homogeneous disease patterns that characterise the clusters found by LCA within this cohort, where most of the population (around 60%) were defined by only three clusters in both males and females.

One of the biggest challenges faced by individuals with MLTC is the need to navigate care from multiple specialists^7^, often leading to inefficiencies and fragmented care, risk of medication errors and eventually poor health outcomes^33^. Specialised clusters could inform strategies to improve person-centred and integrated care. For example, clustering could support the co-location of specialty clinics or foster co-working of specialists managing conditions within the same cluster. This approach could reduce the number of different services a patient needs to visit leading to improved care integration^34^. Given the strong clustering of cardiometabolic conditions, these represent a prime target for strategies to improve care integration, with various models of multi-disciplinary working recently described^35^.

A strength of this study is the use of a large, contemporary dataset which is representative of the English population^36^. An advantage of primary care data is that historic diagnoses can be entered retrospectively with the date of diagnosis, capturing a more complete view of a person’s disease history than is often available in hospital records. A further strength is that while traditional clustering approaches in healthcare often focus on either patient characteristics or clinical outcomes, LLMs offer an opportunity to combine both, by fine-tuning on outcomes such as mortality or hospital admission. This could enable the generation of clusters that not only reflect patient characteristics but also predict downstream risks. Future research could investigate the relationship between model self-attention and cluster assignment to identify sequences of diseases which are at higher risk of adverse health outcomes. Simulated interventions can also be explored by modifying patient histories (e.g. adding medication at a specific time point or delaying a diagnosis), informing personalised lifestyle, or treatment recommendations and drug repurposing.

Some limitations also need to be acknowledged. Although the prevalence of many common diseases in CPRD is similar to other national data sources^37^, for conditions such as cancer, diagnosis recording is less complete than estimates derived from gold standard sources such as cancer registry data^38^. The presence or absence of a diagnostic code in GP EHR data is also influenced by factors independent of a person’s health, such as GP practice organisational coding policies and financial incentives, potentially biasing model learning towards conditions which are recorded more frequently^39^. To mitigate this, we leveraged a broad range of information available in EHRs, including prescription codes, which are well-recorded due to electronic prescribing. This approach allows the model to identify relevant patterns, even when data are missing from a single modality. Additionally, ethnicity data were only available for a subset of the studied population, and certain ethnicities may be under-recorded, although CPRD GOLD has been shown to be broadly representative of the UK population^40^. We summarised continuous diagnostic tests as binary outcomes (e.g. abnormal C-reactive protein levels) which may result in loss of information. Future work could explore use of additional embedding layers that can provide information on continuous variables, such as blood pressure readings. While we presented individual diseases prevalent in each cluster, the underlying embeddings also captured disease sequences over time. Future work could explore whether common diseases trajectories differ between clusters that otherwise appear similar. Additionally, we included individuals with records at age 50, thus conditioning on an individual surviving to this age. This may limit the generalisability of results to younger populations who die before age 50. Finally, in this study we have utilised several internal- and cross- validation methods to demonstrate the robustness of the clusters to our cohort. However, external validation using an independent dataset in future would strengthen the external validity to other populations.

This study highlights the power of LLM-based patient clustering for understanding multimorbidity patterns across age, gender and socioeconomic groups. Our findings stress the need for patient-centred rather than disease-centred interventions and indicate valuable opportunities to explore shared disease mechanisms and potential drug repurposing applications.

## Methods

### Data sources

This retrospective cohort study used data from CPRD GOLD, a nationally representative dataset of ~10 million patients registered to GPs in the UK^36^. CPRD contains comprehensive structured data from EHRs of registered individuals from contributing GP practices which includes diagnoses, prescriptions, laboratory test results and referrals. Data are also linked to a composite measure of socioeconomic deprivation, the Index of Multiple Deprivation (IMD) at patient level, which is grouped into quintiles^41^. Secondary care records are also available for individuals eligible for linkage to Hospital Episode Statistics (HES) and Office of National Statistics (ONS) which includes diagnoses and laboratory test results for hospitalised patients, and death registration data. CPRD GOLD defines the gender (male or female) of an individual as recorded by the healthcare provider. The documented ethnicity of individuals was identified using SNOMED CT codes indicative of ethnicity within CPRD for individuals (Supplementary Table 1) with at least one ethnicity code. Ethnicity was described using five categories as recorded in standard NHS sources: White, South Asian, Black, Mixed and Other^42^. CPRD has established quality assurance through validation processes and the inclusion of GP practices meeting predefined research standards^36^.

### Eligibility

We included all individuals aged 50 years and over who were registered to a participating GP practice for at least 3 years on 31st December 2022.

### Disease definitions

We included 54 chronic conditions and three risk factors within our definition of MLTC which were selected based on the combined list from the Global Burden of Disease Study and a recent Delphi consensus study on MLTC^43,44^. We used available ICD-10 code lists from these studies (Supplementary Table 2) and refined each code list to better capture clinically relevant or ambiguous conditions. For CPRD data, which uses SNOMED-CT codes, ICD-10 codes were mapped to SNOMED CT using the 2022 release of SNOMED-to-ICD map by the NIH Unified Medical Language System to generate a SNOMED CT codelist for each chronic condition and risk factor^45^. Therefore, clinical diagnoses were defined by the presence of either an ICD-10 or SNOMED CT for each condition.

### Longitudinal patient sequences of electronic health records

The study outline for generating a patient embedding, which is the high-dimensional vector that represents a patient’s medical history and MLTC patterns, is shown in Fig. 1. For each patient, a chronological sequence of their medical history was constructed using clinical diagnoses, symptoms, medication prescriptions and laboratory test results. We utilised a total of 3776 different diagnostic codes (ICD-10 codes and medication groups such as ACE inhibitors) to create the patient sequences. With each diagnosis or medication event, we pair the event with additional patient information including the age of the patient when the event occurs, the calendar year the event occurs, the gender of the patient and a visitation number, which is a counter for the number of unique healthcare utilisations each individual has recorded in the chronological sequence based on pooled 1-month period intervals.

Clinical diagnoses included all conditions encoded at the highest level of the ICD-10 hierarchy across all diseases or health conditions chapters (Chapters A to S), and all SNOMED-CT codes which mapped to an ICD-10 code within these chapters. Primary care records of interest were mapped to ICD-10 codes prior to inclusion chronologically in each patient sequence and records with no mapping to an ICD-10 code were excluded. ICD-10 codes that occurred in less than 100 individuals within the entire study were excluded to reduce vocabulary size. Symptoms and laboratory tests were included chronologically using ICD-10 Section R and a manually curated list of SNOMED-CT codes which encode indicators of abnormal test results. Products within the prescription records were first mapped to medication groups based on the primary function of the product (Supplementary Table 3) and subsequently included chronologically in each patient sequence. To reduce the chance of continuous prescriptions dominating each patient sequence, only the first instance of mapped medications prescribed monthly were included in the chronological sequences with a 6-month gap in prescription required before the re-addition of the medication code to the sequence. For mapped medications not prescribed monthly, all prescription events were included chronologically in each patient sequence.

### Generating patient representations

To generate a quantitative representation of each patient’s medical history to be utilised for clustering, we developed a transformer architecture, named EHR-DeBERTa, which can read the chronological sequence of a patient’s medical history, created as previously described, to generate a 768-dimensional vector that encodes all of the diagnosis and medication relationships identified by the model, called a patient embedding. The patient embedding allows us to compare patient similarities by exploring the distance between each patient’s embedding, as identified by EHR-DeBERTa. Previous work applied to structured diagnostic codes in EHR data have used BERT (Bidirectional Encoder Representations from Transformers) architectures, which are a transformer-based deep learning models used for natural language processing^16–18^. Here, we utilise a Decoding-enhanced BERT with disentangled attention (DeBERTa) architecture with a maximum sequence length of 64 tokens, which employs disentangled attention to represent words as two separate vectors (content and position) along with absolute word position embeddings, shown to perform better than the original BERT models in learning more complex relationships in several language modelling tasks^46^. To improve the model’s ability to learn the relationships between events, we included embedding layers for patient age and calendar year at each event, gender of the individual and visit number of the event^16^. To train the EHR-DeBERTa model we utilised the same ELECTRA-style pre-training published with DeBERTa v3, followed by fine-tuning using Difference-based Contrastive learning for Sentence Embeddings (DiffCSE) to ensure robust and improved patient representations^47,48^.

Our ELECTRA style pre-training involved training two models simultaneously using a replaced token detection (RTD) task by firstly masking 15% of clinical events within a sequence and utilising an EHR-DeBERTa model, named the generator, to predict the masked code. The sequence created by the generator with the masked events replaced was then fed into a second EHR-DeBERTa model, called the discriminator, which predicted if each code in the sequence was part of the original sequence or if it has been replaced by the generator. Both models are updated at the same time, utilising a dis-entangled clinical event embedding between the discriminator and generator implemented in the DeBERTa v3 procedure to improve model training. We used the same hyperparameters as the small DeBERTa v3 model consisting of 6 layers in the discriminator and 3 layers in the generator, as described by the original authors, with a maximum sequence length of 64 tokens^46^. In doing this pre-training procedure, the generator builds up a vector representation of each clinical event within its vocabulary based on each clinical events relationship with other events and their interactions with age, sex, calendar year and visit number. The models were trained for a total of 100 epochs to ensure convergence using dynamic masking as suggested by RoBERTa with a 15% of masking rate^49^. Training took a total of 1 week using a single NVIDIA Quatro RTX 8000.

We subsequently fine-tuned the pre-trained EHR-DeBERTa generator on the same training set of samples using DiffCSE which is an equivariant contrastive learning method that ensures the generated patient representations are insensitive to certain types of event alterations within patient sequences, such as swapping a single clinical event to an event which has almost no difference to patient trajectory, and sensitive to other types of event alterations that would cause a drastically different trajectory, considered ‘harmful’ alterations. This was performed using the pretrained EHR-DeBERTa generator model with temperature = 0.05 and lambda = 0.005, as suggested by the original authors, for 5 epochs.

Patient embeddings were generated for the entire cohort by feeding each longitudinal patient sequence, including all paired covariate sequences, one by one through the DiffCSE fine-tuned EHR-DeBERTa model to generate one patient embedding for each patient.

### Generating gender-specific patient clusters

To generate gender-specific patient clusters, we stratified the cohort by gender and applied the clustering pipeline to the patient embeddings of each stratum separately. This involved identification of the optimal number of clusters for each gender followed by K-Means clustering using the optimal number of clusters.

### Cluster validation

We carried out 3 approaches to validate our clusters: clinical plausibility, cluster stability across subsamples and cluster stability across time^50^.

To check that the model was learning clinically meaningful relationships between conditions, we evaluated the outputs with reference to clinically established disease relationships produced in earlier work^32^. We did this by calculating the cosine similarity between pairs of clinically established co-morbidities (Supplementary Table 4) within the clinical code input embeddings of the fine-tuned model.

Robust identification of the optimal number of clusters for each gender was performed by bootstrapping agglomerative hierarchical clustering on 1% subsamples of each stratum across 500 repeats. To ensure subsampling was representative of the entire strata cohort, before subsampling we clustered the entire cohort into 1000 K-Means clusters and for each bootstrapping step we utilised these clusters with random stratified sampling to sample equally from each cluster. Optimal cluster numbers were identified using the mean and standard deviation of the silhouette score across all replicates, with the mean and standard deviation of the Davies-Bouldin and Calinski-Harabasz score used for corroboration along with the dendrograms generated by the agglomerative hierarchical clustering^51^.

After identification of the optimal number of clusters, we performed bootstrapping K-Means clusters on 25% of subsamples of each stratum across 100 replicates. To ensure stability of the final K-Means clusters, we matched each of the replicate K-means centroids to our final K-means centroids generated on the entire population with a 1-to-1 relationship using the Hungarian algorithm based on cosine distances between centroids^52^. After matching the replicates, we calculated the median cosine distance between the matched bootstrapped centroids and each of our final centroids (Supplementary Table 6).

We generated patient embeddings for all participants after splicing their medical records at specific ages, from age 50–100 in 1-year intervals. Individuals that were not registered or had no records after each specific age were not included within each 1-year interval. For example, if we only had records of a participant up to and including age 64, they were excluded from the age 65 cohort. By implementing this process, we generated a single patient embedding for each individual at every year of life based on their medical history up to that age. Using the age-specific patient embeddings, we assigned individuals to one of our clusters at each eligible age based on distance to K-means centroid. Based on cluster assignment, we calculated the median cluster cohesion (cosine distance to centroid) within all our clusters across every age and compared this to the cluster cohesion of our final clusters (Supplementary Table 6).

### Cluster evaluation

We defined the clusters in terms of clinical and socio-demographic factors by testing the statistical association of cluster assignment. Associations with continuous variables were tested using 1-vs-all *t*-tests, binary variables were tested using Chi-squared tests (Supplementary Table 7). In addition, we calculated cluster specific disease-frequency-inverse patient frequency (c-DF-IPF), a method adapted from the natural language processing method of term-frequency-inverse document frequency (TF-IDF)^53^, to create cluster specific weighted disease frequency. DF-IPF reduces the weight of common diseases within the population and emphasises the diseases that are frequent within a cluster, but rare across all clusters. This allows better identification of the important diseases for a cluster based on local (cluster) importance and global (population) infrequency.

To generate c-DF-IPF, we initially calculate the global importance of diseases across the population using the entire corpus. Let $P$ represent the entire patient cohort, we apply DF-IPF (Eq. 1):

1 $${DFIPF}=\frac{{n}_{d,p}}{{\Sigma }_{{d}^{{\prime} }}{n}_{{d}^{{\prime} },p}}\cdot \log \left(\frac{\left|P\right|}{\left|\left\{p\epsilon P:d\epsilon p\right\}\right|}\right)$$

where ${n}_{d,p}$ is the presence of disease $d$ in patient record $p$ and ${\Sigma }_{{d}^{{\prime} }}{n}_{{d}^{{\prime} },p}$ is the total number of diseases within the patient’s record. To calculate cluster specific importance, we averaged the DF-IPF of individuals within each cluster and scaled these using cluster specific inverse patient frequencies, ${IP}{F}_{i}$ (Eq. 2):

2 $$c-{DFIPF}={DFIP}{F}_{i}\cdot {ID}{F}_{i}={DFIP}{F}_{i}\cdot \log \left(\frac{\left|K\right|}{\left|d\epsilon K\right|}\right)$$

where ${DFIP}{F}_{i}$ represents the average cluster importance of cluster *i*, ${|K|}$ is the total number of clusters and ${|d}\epsilon {K|}$ is the number of clusters disease $d$ is present within.

We additionally calculated the Observed-Expected ratios and Exclusivity of each LTC (Supplementary Table 8 and Supplementary Table 9). Clusters were named and grouped clinically according to the common patterns of diseases within them, focusing on those conditions that varied significantly between clusters. Within each category, clusters were then numbered in descending order of prevalence. Ethnicity and social deprivation associations to clusters were only performed in individuals with an ethnicity SNOMED record or linkage to IMD, respectively. To compare our method to other commonly used clustering techniques, we additionally performed LCA on the male and female cohorts separately using the optimal number of clusters across all LTCs for comparison and the optimum number of classes was identified using AIC and BIC.

We used Python version 3.11.5 and Pytorch version 2.0.1 to create and train the LLM models. Statistical analysis and data processing were performed using sci-kit learn version 1.3.2. Ethical approval for CPRD data and linkage to HES data was granted by CPRD’s Research Data Governance Process on 23rd September 2021 (protocol reference: 20_000209). Patient informed consent is unnecessary as the data is anonymised by CPRD who have ethics approval from the Health Research Authority to support research using anonymised patient data (21/EM/0265).

## Supplementary information

Supplementary Figure.

Supplementary Table.

## Data Availability

To maintain patient anonymity, data are not publicly available but can be requested from CPRD for users meeting certain requirements as described here: https://cprd.com/research-applications.

## Code availability

The EHR-DeBERTa model was based on the original implementation found at https://github.com/microsoft/DeBERTa. DiffCSE fine-tuning was implemented as an adaptation from the original code found at: https://github.com/voidism/DiffCSE.
