# Supplementary material for: Identifying clusters of people with Multiple Long-Term Conditions using Large Language Models: a population-based study
Source: NPJ Digit Med. 2025 Jul 17;8:453. doi: 10.1038/s41746-025-01806-9 (PMC12271452; doi:10.1038/s41746-025-01806-9)

**Supplementary Figure 1. Age distribution of patient clusters**. The age of individuals at their first and last recorded diagnosis within our cohort within each cluster.


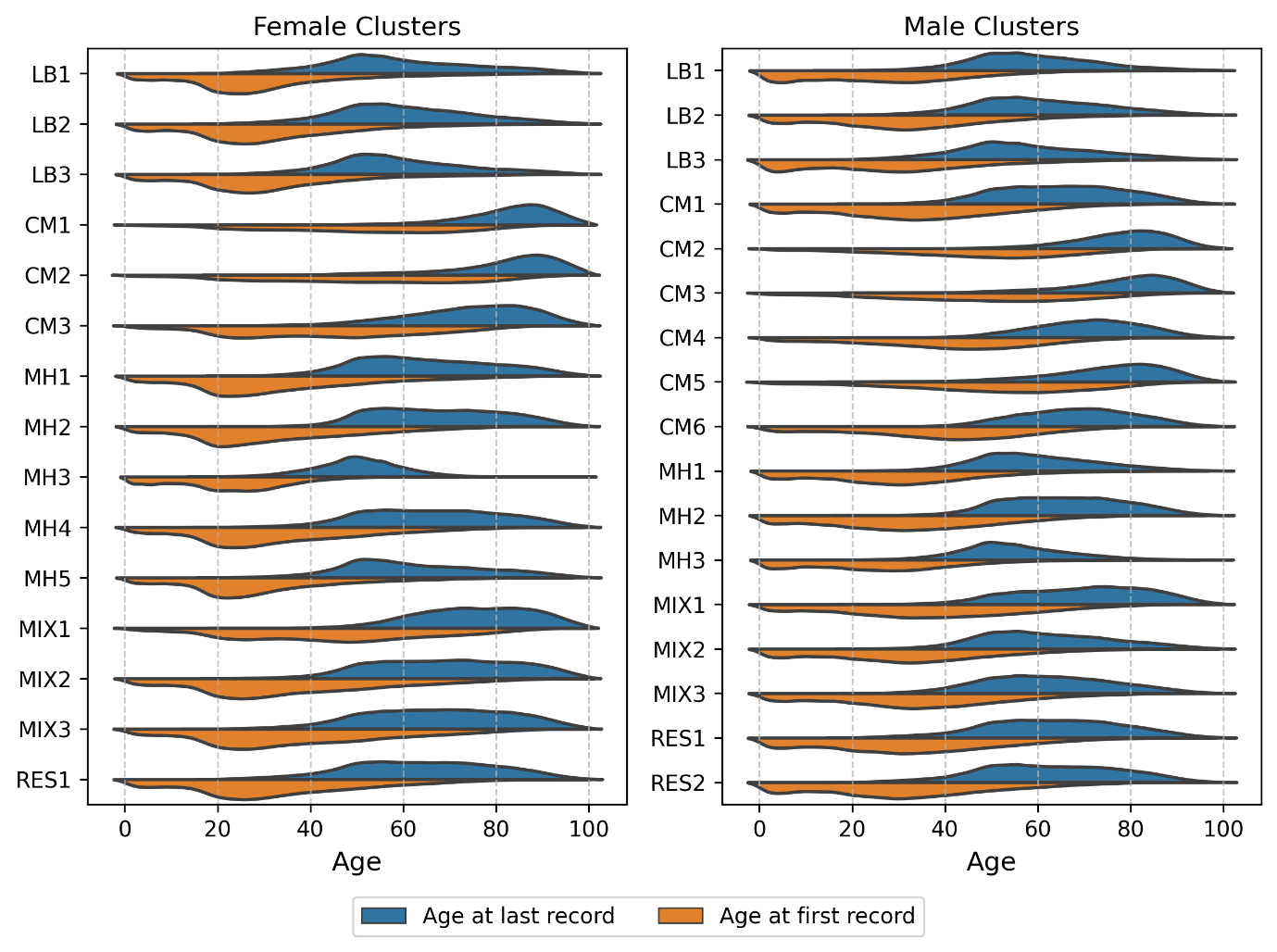


**Supplementary Figure 2: Cluster disease similarity.** A heatmap of weighted disease frequency (c-DF-IPF) cosine similarity between each of the male and female clusters.


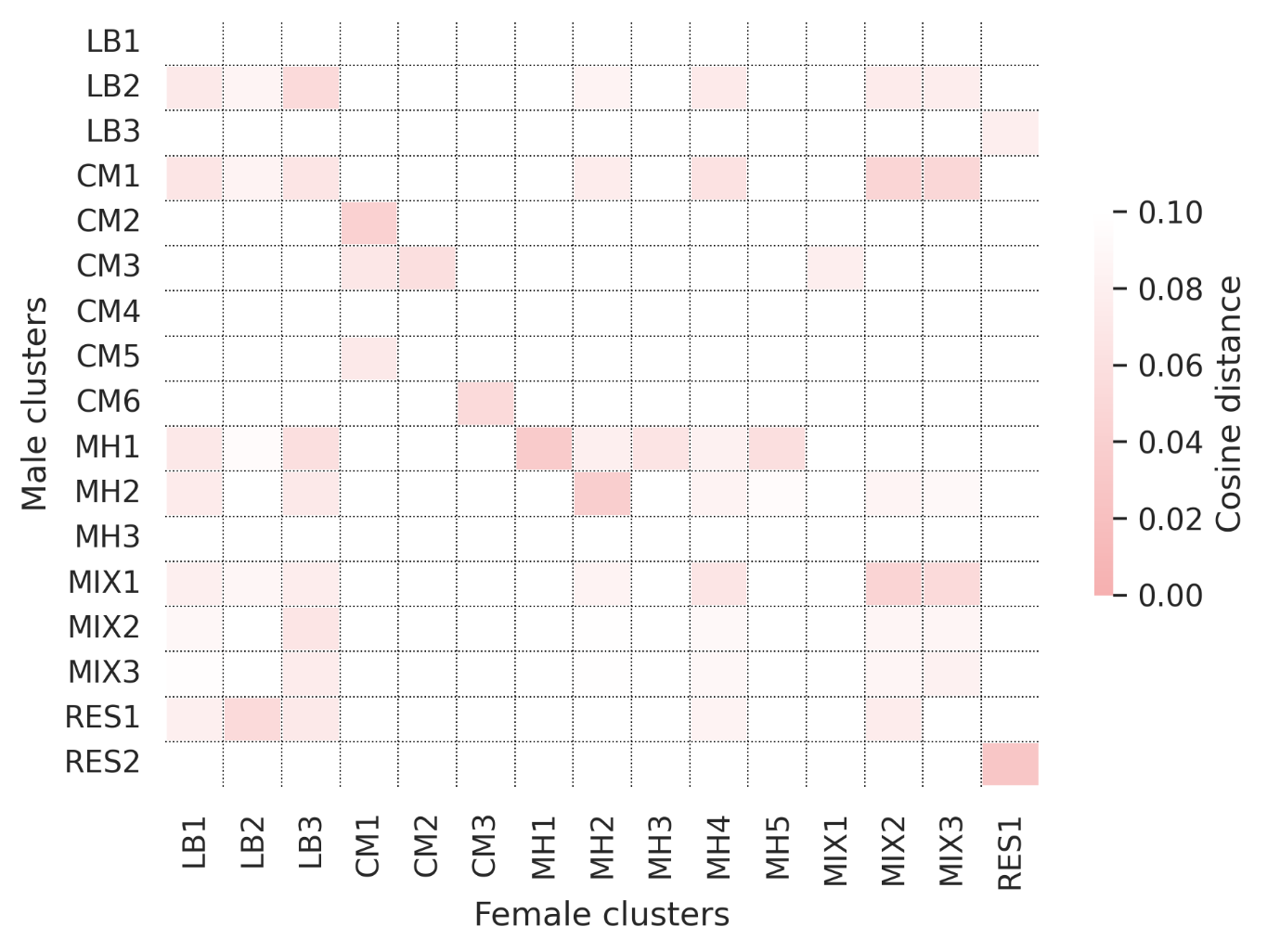

Supplement: Supplementary file 1 — Supplementary Figure. [file 41746_2025_1806_MOESM1_ESM.docx]
